# Fossil flowers from the early Palaeocene of Patagonia, Argentina, with affinity to Schizomerieae (Cunoniaceae)

**DOI:** 10.1093/aob/mcx173

**Published:** 2018-01-04

**Authors:** Nathan A Jud, Maria A Gandolfo, Ari Iglesias, Peter Wilf

**Affiliations:** 1 L. H. Bailey Hortorium, Plant Biology Section, School of Integrative Plant Science, Cornell University, Ithaca, NY, USA; 2 Universidad Nacional del Comahue, Instituto de Investigaciones en Biodiversidad y Ambiente INIBIOMA-CONICET, San Carlos de Bariloche, Rio Negro, Argentina; 3 Department of Geosciences, Pennsylvania State University, University Park, PA, USA

**Keywords:** Danian, palaeobotany, Patagonia, Gondwana, Salamanca Formation, Peñas Coloradas Formation, K-Pg recovery

## Abstract

**Background and Aims:**

Early Palaeocene (Danian) plant fossils from Patagonia provide information on the recovery from the end-Cretaceous extinction and Cenozoic floristic change in South America. Actinomorphic flowers with eight to ten perianth parts are described and evaluated in a phylogenetic framework. The goal of this study is to determine the identity of these fossil flowers and to discuss their evolutionary, palaeoecological and biogeographical significance

**Methods:**

More than 100 fossilized flowers were collected from three localities in the Danian Salamanca and Peñas Coloradas Formations in southern Chubut. They were prepared, photographed and compared with similar extant and fossil flowers using published literature and herbarium specimens. Phylogenetic analysis was performed using morphological and molecular data.

**Key results:**

The fossil flowers share some but not all the synapomorphies that characterize the Schizomerieae, a tribe within Cunoniaceae. These features include the shallow floral cup, variable number of perianth parts arranged in two whorls, laciniate petals, anthers with a connective extension, and a superior ovary with free styles. The number of perianth parts is doubled and the *in situ* pollen is tricolporate, with a surface more like that of other Cunoniaceae outside Schizomerieae, such as *Davidsonia* or *Weinmannia*.

**Conclusions:**

An extinct genus of crown-group Cunoniaceae is recognized and placed along the stem lineage leading to Schizomerieae. Extant relatives are typical of tropical to southern-temperate rainforests, and these fossils likely indicate a similarly warm and wet temperate palaeoclimate. The oldest reliable occurrences of the family are fossil pollen and wood from the Upper Cretaceous of the Antarctica and Argentina, whereas in Australia the family first occurs in upper Palaeocene deposits. This discovery demonstrates that the family survived the Cretaceous–Palaeogene boundary event in Patagonia and that diversification of extant lineages in the family was under way by the earliest Cenozoic.

## INTRODUCTION

The lower Palaeocene (Danian) deposits that crop out in central Patagonia, Argentina, yield exquisitely preserved plant remains (Berry, 1937; Romero, 1968; Petriella, 1972; Archangelsky, 1973; Petriella and Archangelsky, 1975; Archangelsky and Zamaloa, 1986; Brea *et al.*, 2005, 2008; Iglesias *et al.*, 2007; Barreda *et al.*, 2012; Futey *et al.*, 2012; Donovan *et al.*, 2016; Ruiz *et al.*, 2017). These fossils provide some of the oldest reliable Danian records of taxa that survived the end-Cretaceous extinction event in the Southern Hemisphere. Among these fossils are delicate flowers with well-preserved features that permit high confidence in their systematic identifications (e.g. Jud *et al.*, 2017).

Among the most common reproductive macrofossils in the Salamanca Formation are the flowers presented here. They have a suite of features corresponding to the family Cunoniaceae R.Br. The Cunoniaceae are trees and shrubs comprising 27 extant genera and over 300 species found throughout tropical and temperate habitats in the Southern Hemisphere (Good, 1974; Bradford *et al.*, 2004). They are characterized by opposite or whorled compound (rarely simple) leaves and bicarpellate or tetracarpellate ovaries, and many produce dicolporate pollen (Hill and Macphail, 1983; Bradford *et al.*, 2004). The oldest evidence of Cunoniaceae is fossil pollen and wood from sites that date to the Late Cretaceous (Santonian–Maastrichtian) of Antarctica (Cranwell, 1959; Askin, 1992; Poole *et al.*, 2000, 2003), and Maastrichtian pollen (Baldoni and Askin, 1993) from Patagonia. The family was extirpated from Antarctica sometime after the middle Eocene (Cranwell, 1959; Askin, 1997; Cantrill and Poole, 2012; Tosolini *et al.*, 2013) but persisted in South America, Africa and Australia, ultimately spreading to Central America and Indomalesia (Bradford *et al.*, 2004).

In this contribution, we describe adpressed flowers with preserved sepals, petals, stamens with *in situ* tricolporate pollen and ovaries with two to four styles from the early Palaeocene (Danian) Salamanca and Peñas Coloradas Formations. The flowers have a combination of character states found in Cunoniaceae, and petals like those of Schizomerieae. To understand the evolutionary significance of these fossils we used parsimony analysis of morphological and molecular data. We discuss the implications of the results for understanding the composition of the earliest Palaeocene floras of Southern South America and the survival of Gondwanan plant lineages.

## MATERIALS AND METHODS

**Fig. 1:**
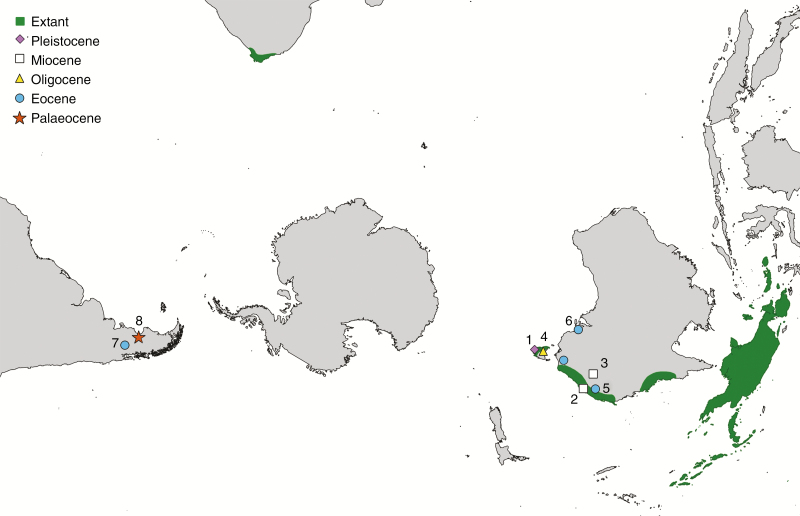
Map showing the distribution of extant Schizomerieae and fossil occurrences of the tribe. Fossils shown are (1) *Anodopetalum biglandulosum* (Jordan *et al.*, 1991); (2) *Ceratopetalum westermannii* (Barnes and Hill, 1999); (3) *Ceratopetalum priscum* (Holmes and Holmes, 1992); (4) *Schizomeria tasmaniensis* (Carpenter and Buchanan, 1993); (5) *Ceratopetalum wilkinsonii* (von Ettingshusen, 1888; Holmes and Holmes, 1992; Barnes and Hill, 1999); (6) *Ceratopetalum maslinensis* (Christophel and Blackburn, 1978; Barnes and Hill, 1999); (7) *Ceratopetalum edgardoromeroi* (Gandolfo and Hermsen, 2017); and (8) *Lacinipetalum spectabilum* Jud, Gandolfo, Iglesias & Wilf, gen et sp. nov.

We used traditional survey and excavation methods to collect plant fossils from the study area. A total of 113 fossils of the species studied here were collected from three sites in Danian (early Palaeocene) deposits of the San Jorge Basin, southern Chubut Province, Argentina (Fig. 1, Table 1). The fossils were collected over four field seasons (2005, 2009, 2011 and 2012). The stratigraphical and geochronological framework and facies interpretations of these sites are detailed in Clyde *et al.* (2014) and Comer *et al.* (2015), which also list GPS coordinates. Other locality data are available at the Museo Paleontológico Egidio Feruglio (MEF), Trelew, Chubut, Argentina, where the specimens are curated. Most of the flowers (107 specimens) were collected from the Palacio de los Loros-2 (PL-2) site in the Salamanca Formation; this site is correlated to geomagnetic polarity Chron C28n (Clyde *et al.*, 2014; Comer *et al.*, 2015), which spans 64.67–63.49 Ma (Gradstein *et al.*, 2012). The PL-2 site yields a parautochthonous assemblage of leaves and reproductive structures preserved in a grey shale interpreted as a tidally influenced channel-fill (Iglesias *et al.*, 2007; Comer *et al.*, 2015). Four specimens were collected from the Palacio de los Loros-5 (PL-5) locality, which is slightly more coarse-grained than PL-2 and nearly 1 km away, but also interpreted as a tidally influenced channel-fill deposit from Chron C28n (Iglesias *et al.*, 2007; Comer *et al.*, 2015). Two specimens came from the fluvio-volanic Las Flores locality (LF), which is in the late Danian Peñas Coloradas Formation. The LF locality is correlated to Chron C27n (Clyde *et al.*, 2014; Comer *et al.*, 2015), which spans 62.52–62.22 Ma (Gradstein *et al.*, 2012), and the fossils are preserved in reddish, fissile mud that is wedged between cross-bedded sets of poorly sorted, cross-bedded sandstone. The LF locality is interpreted as a fluvial channel-fill deposit (Comer *et al.*, 2015).

**Table 1.** Summary of megafossil occurrences accepted here as Schizomerieae

	Species	Organ	Age	Site(s)	Source
1	*Anodopetalum biglandulosum* A. Cunn. ex Hook F.	Leaves	Pleistocene	Melaleuca Inlet	Jordan *et al.*, 1991
2	*Ceratopetalum westermannii* R.W. Barnes & R.S. Hill	Fruit	Miocene	Elands	Barnes and Hill, 1999
3	*Ceratopetalum priscum* W.B.K. Holmes & F.M. Holmes	Flower	Miocene	Chalk Mountain Fm.	Holmes and Holmes, 1992
4	*Schizomeria tasmaniensis* R.J. Carpenter & A.M. Buchanan	Flower	Oligocene	Cethana	Carpenter and Buchanan, 1993
5	*Ceratopetalum wilkinsonii* (Ett.) W.B.K. Holmes & F.M. Holmes emend. R.W. Barnes & R.S. Hill	Flower	Eocene	Vegetable Creek	von Ettingshausen, 1888; Holmes and Holmes, 1992; Barnes and Hill, 1999
6	*Ceratopetalum maslinensis* R.W. Barnes & R.S. Hill	Fruit	Eocene	Maslin Bay South	Barnes and Hill, 1999; Alley, 1998
7	*Ceratopetalum edgardoromeroi* M.A. Gandolfo & E.J. Hermsen	Fruit	Eocene	Laguna del Hunco	Gandolfo and Hermsen, 2017
8	*Lacinipetalum spectabilum* gen. et sp. nov.	Flowers	Palaeocene	PL-2, PL-5, LF	This study

The fossil flowers were prepared by degauging. Images of macroscopic features were captured with a Canon EOS 7D DSLR camera, and microscopic details were photographed with a Nikon DS Fi1 camera mounted on a Nikon SMZ1000 stereoscope at the MEF. Epifluorescence microscopy revealed the presence of pollen grains in the anthers, among the hairs on the compressed ovaries, and on the sepals. Fossil pollen grains were observed under an FEI Quanta 200 environmental scanning electron microscope at the Materials Characterization Laboratory, Pennsylvania State University (PSU, PA, USA). Pollen grains from extant members of Schizomerieae were mounted on scanning electron microscope stubs and sputter-coated with gold/palladium for observation in a Jeol NeoScope JCM-5000 scanning electron microscope at the Paleontology Research Institute in Ithaca, NY, USA. Images were processed with Adobe Photoshop CC 2017 (San Jose, CA, USA). The fossil specimens are curated in the Paleobotanical Collection of the Museo Paleontológico Egidio Feruglio (MPEF-Pb), Trelew, Chubut, Argentina. We compared them with other relevant fossils using descriptions and illustrations available in the literature and with herbarium specimens (Table 2) housed at the L.H. Bailey Hortorium herbarium (BH) at Cornell University, Ithaca, NY, USA.

**Table 2.** Summary of modern specimens examined

Species	Herbarium voucher
*Ceratopetalum gummiferum* Sm.	BH 081372
*Ceratopetalum gummiferum*	BH 081374
*Ceratopetalum gummiferum*	BH 081376
*Ceratopetalum gummiferum*	BH 095694
*Ceratopetalum gummiferum*	BH 095695
*Ceratopetalum apetalum* D. Don.	BH 081370
*Ceratopetalum apetalum*	BH 081371
*Ceratopetalum apetalum*	BH 081373
*Schizomeria ovata* D. Don	BH 081381
*Schizomeria ovata*	BH 081382
*Schizomeria ovata*	BH 095714
*Schizomeria ovata*	BH 095715
*Schizomeria sp.* D. Don.	BH 095716
*Schizomeria sp.*	BH 095717
*Schizomeria sp.*	BH 095718
*Schizomeria sp.*	BH 095719
*Anodopetalum biglandulosum* (Hook.) Hook f.	BH 081380
*Platylophus trifoliatus* (L.f.) D. Don.	BH 046253
*Platylophus trifoliatus*	BH 053995
*Platylophus trifoliatus*	BH 053996
*Davidsonia pruriens* F. Muell.	BH 123703
*Davidsonia pruriens*	BH 123704

We created a modified matrix of morphological characters for Schizomerieae and closely related Cunoniaceae to integrate the fossil taxon into a phylogenetic framework. We started with the matrices developed by Bradford and Barnes (2000) and Rozefelds and Barnes (2002). We modified or created 20 of the morphological characters and scored them using direct observation of herbarium specimens and the results of prior studies (Dickison, 1980, 1984; Barnes and Rozefelds, 2000; Matthews *et al.*, 2001; Matthews and Endress, 2002; Rozefelds and Barnes, 2002). The new morphological matrix comprises eight terminals (outgroup: *Davidsonia*; ingroup: *Anodopetalum*, *Platylophus*, two species each of *Ceratopetalum* and *Schizomeria*, and the Patagonian fossil taxon) and 63 morphological characters. The character descriptions and matrix are available online at the MorphoBank website (https://www.morphobank.org; project P2533, Schizomerieae phylogeny). We also obtained molecular data for the extant terminal taxa. Previously published *rbcL* and *trnL-trnF* sequences were downloaded from GenBank (Bradford and Barnes, 2001; Sweeney *et al.*, 2004; accession numbers are listed in Table 3 and and the aligned sequence data are provided in Supplementary Data Appendix S1). The sequences were aligned using the MUSCLE program (Edgar, 2004) and implemented in AliView (Larsson, 2014) under default parameters.

**Table 3.** Summary of the genetic data and GenBank accession numbers used to conduct the phylogenetic analysis. All taxa contributed morphological data

Taxon	*rbcL*	*trnL-F*	*trnL* intron	*trnL-F* igs
*Ceratopetalum gummiferum*	L01895.1	–	AF299176.1	AF299229.1
*Ceratopetalum apetalum*	KM895900.1	–	NA	NA
*Schizomeria ovata*	–	–	AF299178.1	AF299231.1
*Schizomeria serrata*	JX236031.1	JX236028.1	–	–
*Anodopetalum biglandulosum*	AF291932.1		AF299175.1	AF299228.1
*Platylophus trifoliatus*	AF291933.1		AF299177.1	AF299230.1
*Davidsonia pruriens*	AF206759.1	KC428488.1	–	–
*Lacinipetalum* *spectabilum* gen. et sp. nov.	NA	–	NA	NA

Phylogenetic relationships were inferred first from the combined nucleotide and morphological data using maximum parsimony (MP) implemented in the phylogenetic software TNT (Goloboff *et al.*, 2008) spawned through ASADO (Nixon, 2008). To minimize *a priori* assumptions about the relative value of the characters, all characters were unweighted and unpolarized, and multistate characters were unordered. Default values for ratchet, drift, sectorial search and tree fusion were retained. We compared the results of this total evidence approach with an MP analysis of the morphological data alone, an MP analysis of the molecular sequence data alone, and a maximum likelihood analysis of the molecular data alone with a GTRGAMMA model of nucleotide substitution implemented in RAxML (Stamatakis, 2014).

## RESULTS

### Systematics

#### Order.

Oxalidales Heintze 1927.

#### Family.

Cunoniaceae R.Br. 1814.

#### Tribe.

Schizomerieae J.C. Bradford & R.W. Barnes 2001.

#### Genus.

*Lacinipetalum* Jud, Gandolfo, Iglesias & Wilf, gen. nov.

#### Type species.

*Lacinipetalum spectabilum* Jud, Gandolfo, Iglesias & Wilf, sp. nov.

#### Generic diagnosis.

Flowers pedicellate, 8- to 10-merous, actinomorphic, perfect; hypanthium palletiform; sepals lanceolate, inserted at the margin of the hypanthium; petals flabellate, laciniate, and equal or longer than the sepals; petal incision varies from ternate to twice ternate; apices of the petal lobes acute; anthers dorsifixed, versatile, about as long as wide, with two pollen sacs, and with a connective extension that is shorter than the length of the pollen sacs; pollen grains tricolporate, prolate, isopolar; exine homogeneous, punctate; gynoecium superior and syncarpous with two or four erect and free stylodia; ovary pubescent; floral disc filling the hypanthium.

#### Holotype designated here.

**Fig. 2.**
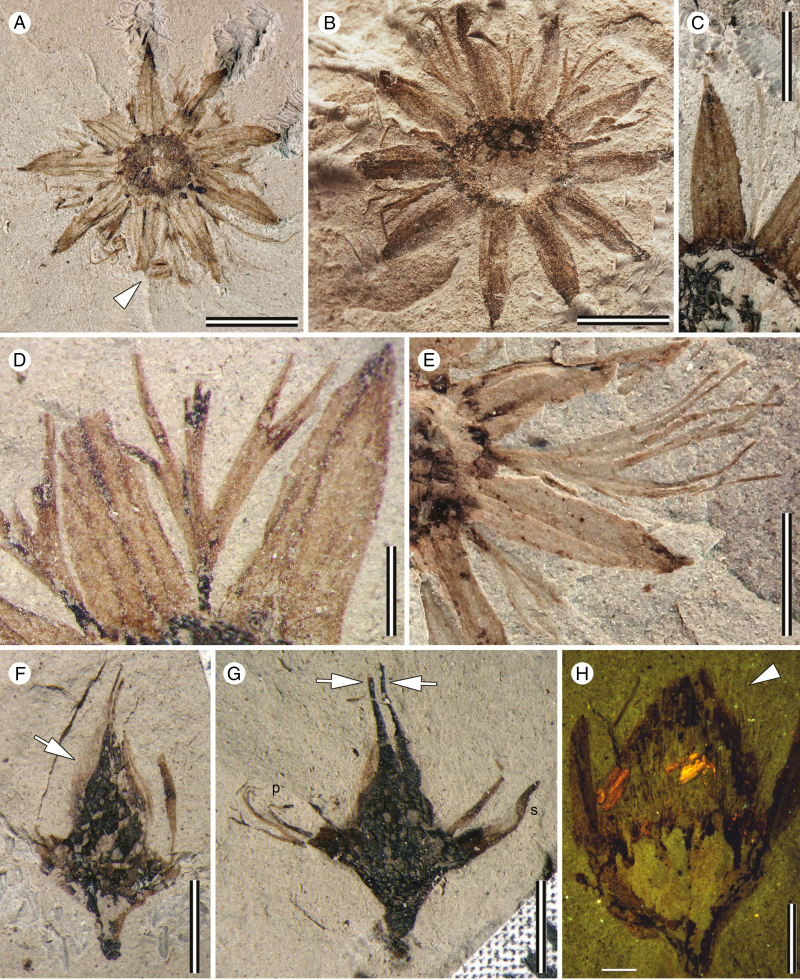
*Lacinipetalum spectabilum* specimens from locality PL-2. (A) Flower in transverse view, showing 9-merous structure, sepals narrow-lanceolate, laciniate petals alternating with sepals, stamens (at arrowhead) and floral disc 2.5 mm across. MPEF-Pb 8423. (B) Flower in transverse view, showing 10-merous structure, sepals narrow-lanceolate, laciniate petals alternating with sepals, stamens not preserved, and floral disc 3.9 mm across. MPEF-Pb 8517. (C) Detail of sepal venation. Note three main parallel veins supplying the sepals and two faint intramarginal veins. MPEF-Pb 8433a. (D) Close-up of a narrow, flabellate petal incised into at least eight secondary segments. MPEF-Pb 8447a. (E) Close-up of a narrow, flabellate petal incised into nine segments. MPEF-Pb 8414a. (F) Flower in longitudinal view, showing stout pedicel, shallow floral cup and superior ovary covered in fine trichomes (at arrow) and two free stylodia. MPEF-Pb 8455. (G) Flower in longitudinal view, showing stout pedicel, shallow floral cup, sepals (s), laciniate petals (p) and two stylodia emerging from the apex of the ovary (at arrows). MPEF-Pb 8444a. (H) Flower in longitudinal view illuminated under epifluorescence. Note the two brightly fluorescing anthers and pubescent surface of the ovule (hairs at arrowhead). MPEF-Pb 8452a. Scale bars: (A, B) = 3.0 mm; (C–E) = 2.0 mm; (F, G) = 2.5 mm; (H) = 1.5 mm.

MPEF-Pb 8423 (Fig. 2A), from Palacio de los Loros-2 (PL-2), Chubut Province, Argentina; upper Salamanca Formation, Chron C28n, early Danian (early Palaeocene).

#### Repository.

Museo Paleontológico Egidio Feruglio.

#### Paratypes.

**Fig. 3.**
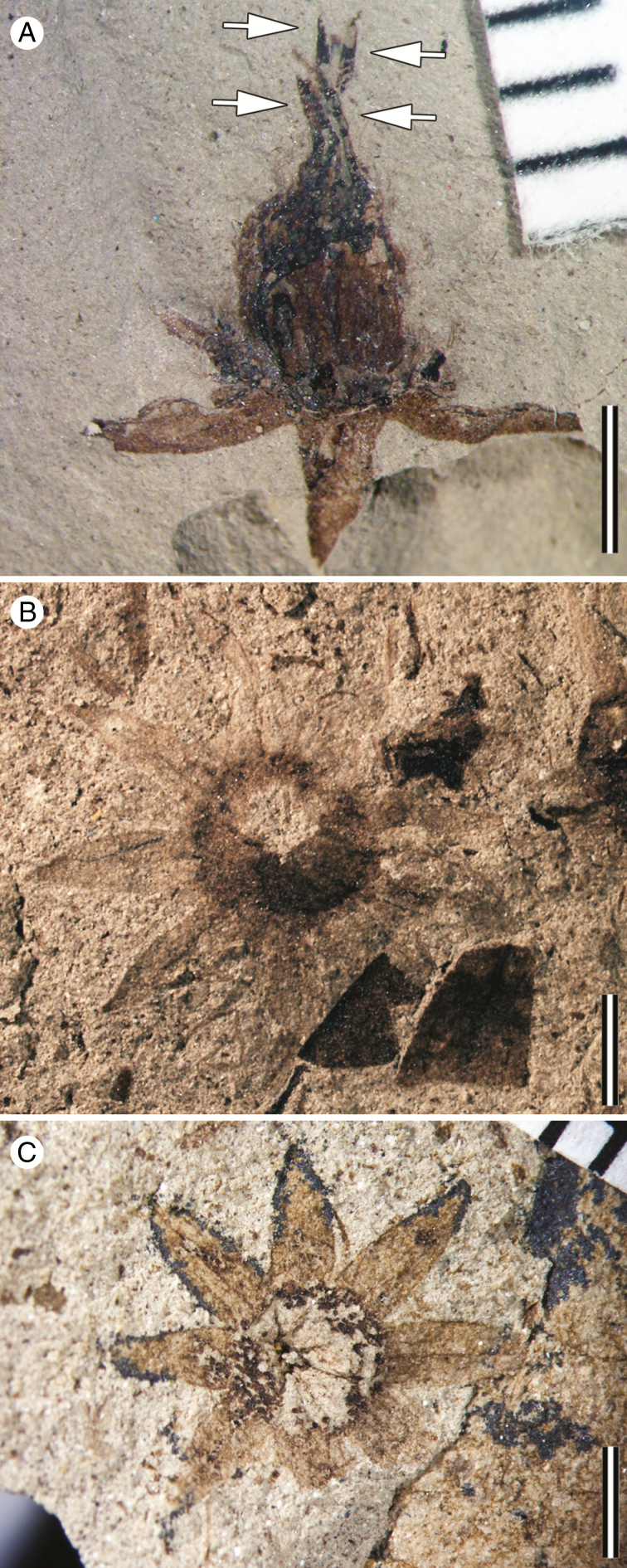
*Lacinipetalum spectabilum* specimens from the PL-2 (A), PL-5 (B) and Las Flores (C) localities. (A) Flower in longitudinal view, showing four stylodia (at arrows). (B) Flower in transverse view, showing nine sepals. MPEF-Pb 9727a. (C) Flower preserved in transverse view, showing 8-merous calyx with sepals supplied by three parallel veins and intramarginal veins. MPEF-Pb 9728. Scale bars: (A–C) = 2.0 mm.

**Fig. 4.**
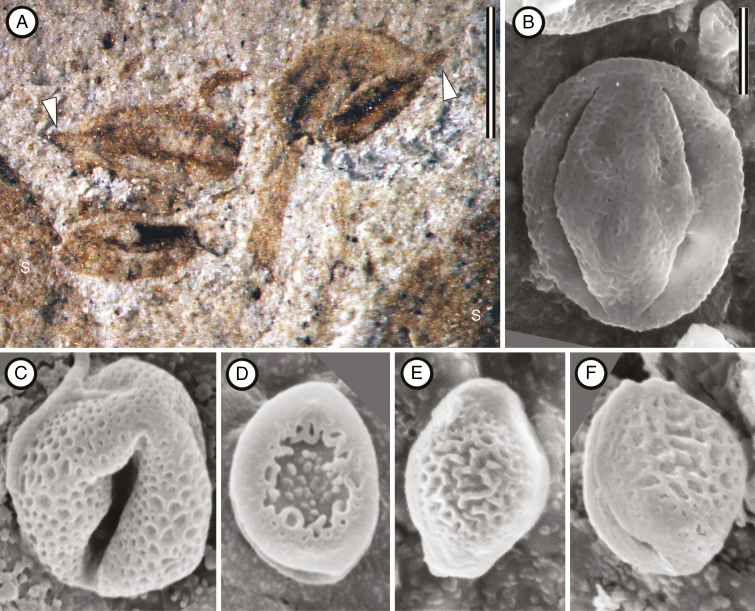
Anther and pollen of *Lacinipetalum spectabilum* (A, B) and Cunoniaceae pollen from modern genera (C–F). (A) Anthers showing the short connective extensions (at arrowheads). MPEF-Pb 8423. s, sepal. (B) Environmental scanning electron microscope (SEM) image of a flattened tricolporate pollen grain with a homogeneous, punctate exine. MPEF-Pb 8452a. (C) SEM image of a tricolporate pollen grain of *Davidsonia pruriens* BH 123703. Note the homogeneous, punctate tectum. (D). SEM image of a dicolporate grain of *Schizomeria ovata* BH 81381. Note heterogeneous tectum. (E) SEM image of dicolporate grain of *Ceratopetalum gummiferum* BH 81374. (F) SEM image of dicolporate grain of *Platylophus trifoliata* BH 46253. Scale bars: (A) = 0.5 mm; (B–F) = 4 µm.

From PL-2 MPEF-Pb 8423 (Figs 2A and 4A), MPEF-Pb 8517 (Fig. 2B), MPEF-Pb 8443a (Fig. 2C), MPEF-Pb 8447a (Fig. 2D), MPEF-Pb 8414a (Fig. 2E), MPEF-Pb 8455 (Fig. 2F), MPEF-Pb 8444a (Fig. 2G), MPEF-Pb 8452a (Figs 2H and 4B), and MPEF-Pb 8463 (Fig. 3A); from PL-5: MPEF-Pb 9727a (Fig. 3B); from LF: MPEF-Pb 9728 (Fig. 3C).

#### Additional material examined.

In addition to the holotype and 11 paratype specimens figured here, we examined a total of 99 other specimens from PL-2 and three from PL-5 in the Salamanca Formation (C28n, early Palaeocene) and one from the Las Flores (LF) locality in the Peñas Coloradas Formation (C27n, early Palaeocene). This material is curated with the types at MEF.

#### Etymology.

The genus is named for the laciniate petals and the specific epithet for the numerous perianth parts.

#### Specific diagnosis.

As for the genus *Lacinipetalum*.

#### Description.

The flowers are perfect and actinomorphic, ~10 mm diameter (8–14 mm), with a shallow floral cup 2.5–4 mm in diameter. The perianth is composed of calyx and corolla, each with eight to ten parts and whorled phyllotaxis. The sepals are valvate and lanceolate (3.5 mm long by 1 mm wide); their bases are broadly attached at the rim of the floral cup and their apices are acute and straight (Fig. 2C). Three parallel major veins supply each sepal; the medial vein is slightly thicker than the two outer veins. In well-preserved specimens, there are also parallel thinner intramarginal veins near the sepal margin and a faint reticulum (Fig. 2C). The petals are alternisepalous, narrow, flabellate and laciniate (Fig. 2A, C–E). The pattern of incision varies from ternate to pedate to twice ternate (three to nine narrow lobes); they are 0.2–0.4 mm wide at the base and 2–5 mm long. Each petal is supplied by a single vein that divides with the lobes (Figs 2C–E and 3C). The androecium comprises <20 stamens; some appear opposite the petals, and in some specimens up to three compressed anthers are visible between the sepals (Fig. 4A). The stamen filaments are slender, ~3 mm long and bear dithecal dorsifixed anthers (Fig. 4A). The anthers are 0.67 mm long and have a connective extension that is ~0.14 mm long (*n* = 3). The shape of the anthers is most consistent with dehiscence along a longitudinal slit (Fig. 4A). The pollen grains found *in situ* are tricolporate and prolate. The colpi taper and almost meet at the poles; the exine is homogeneous and punctate (Fig. 4B). The pollen grains are compressed, 13.3 µm (*n* = 11) long from pole to pole and 9.5 µm across the equator (*n* = 11). The gynoecium is superior and syncarpous (Fig. 2F–H). In most specimens two free styles are visible (Fig. 2F, G), but upon further investigation of several specimens there are in fact four free styles, indicating the tetracarpellate condition (Fig. 3A). The ovary is 2.8–3.4 mm long and covered in acicular trichomes (Fig. 2F–H). The stylodia are at least 2.5–3 mm long, erect, and have indistinct stigmas. At the base of the gynoecium, the flowers appear thickened and often have abundant coalified remains suggestive of a floral disc (Fig. 2A–C, F).

### Phylogenetic analyses

**Fig. 5.**
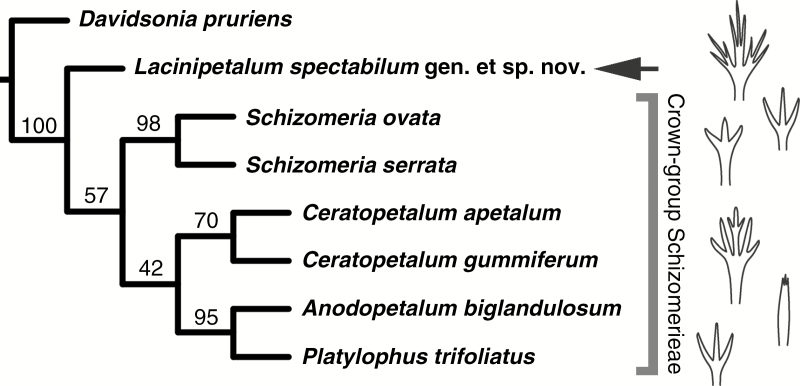
Cladogram showing the results from combined parsimony analysis of the concatenated morphology, *rbcL* and *trnL-trnF* dataset, including the new fossil taxon, whose position is indicated by an arrow. Numbers above the branches are bootstrap values based on an analysis with the subset of 22 parsimony-informative characters.

The concatenated matrix of morphological characters and aligned *rbcL* and *trnL-trnF* sequence data comprises 2610 characters, of which 40 are parsimony-informative (23 morphological and 17 molecular characters). All non-informative characters were omitted from the matrix to optimize subsequent analyses of branch support (i.e. bootstrap support). Parsimony analysis of the combined molecular and morphological dataset (total evidence) yielded a single most parsimonious tree of 59 steps with a consistency index (CI) of 0.76 and a retention index (RI) of 0.70 (Fig. 5). Bootstrap support values for nodes on the tree range from 42 to 100 %. *Lacinipetalum* resolved as sister to Schizomerieae. *Schizomeria* is sister to the other extant genera in the tribe. *Anodopetalum* and *Platylophus* form a clade that is sister to *Ceratopetalum*. Independent parsimony analyses (see Materials and methods section) of the morphological data, complete *rbcL* sequence data and complete *trnL-trnF* sequence data all yielded congruent topologies, as did ML analysis using only molecular data.

## DISCUSSION

### Comparison with extant and fossil taxa

The fossil flowers described here can be identified as Cunoniaceae based on a combination of characters that together are diagnostic of the family. These features include the presence of a shallow floral cup, valvate sepals, flabellate laciniate petals, dorsifixed versatile anthers with a distinct connective extension, the presence of a floral disc, a superior ovary that is hairy and bi(tetra)carpellate, and free stylodia (Hufford and Dickison, 1992; Bradford and Barnes, 2001; Matthews and Endress, 2002; Bradford *et al.*, 2004).

The narrow, laciniate petals in the fossils inspired the initial comparison with Schizomerieae; however, this feature can also be found in the related families Elaeocarpaceae and Connaraceae (Matthews and Endress, 2002). Some of the genera in these families also share other features with the Schizomerieae, such as the shallow floral cup, two to four fused carpels and the occurrence of some within-plant variability in the number of perianth parts. Nevertheless, many features of the androecia of Elaeocarpaceae and Connaraceae are quite different from those of Cunoniaceae. For example, in extant Schizomerieae and the Patagonian fossils the stamens are free, the anthers are short (i.e. the thecae are only about as long as the anther is wide), introrse, dorsifixed and versatile, they have a connective extension, and the thecae dehisce along longitudinal slits. By contrast, in Elaeocarpaceae the anthers are basifixed, much longer than wide, lack a connective extension (except *Sloanea*) and are either apically dehiscent or poricidal (Matthews and Endress, 2002). In Connaraceae, the stamens are congenitally united into a short tube and the anthers lack a connective extension (Matthews and Endress, 2002). Thus, we reject the possibility that these fossils are closely related to Elaeocarpaceae or Connaraceae.

Flowers with two whorls of stamens in which the outer whorl of stamens is opposite to the petals (obdiplostemony) and reduced relative to the inner whorl is typical of the Oxalidales, including Cunoniaceae (Eckert, 1966; Matthews *et al.*, 2001; Matthews and Endress, 2002; Ronse de Craene and Bull-Hereñu, 2016). Since we are confident that *Lacinipetalum* belongs to Cunoniaceae, we interpret the androecium in the fossils as obdiplostemonous as well; however, none of the fossils clearly shows 16–20 stamens. We consider two possible explanations. First, it is possible that most of the anthers are either missing or not visible in the fossils because the filaments are delicate and curled, or they are not preserved. Second, it is possible that the outer whorl was reduced to staminodes or missing, a common occurrence in Oxalidales but rare in extant Schizomerieae (Matthews and Endress, 2002). The first interpretation is supported by the observation that in some specimens there are up to three anthers preserved between the sepals (Fig. 4A), whereas the second interpretation is supported by the observation that anthers are never so numerous as eight or more in any of the specimens examined.

Within the Cunoniaceae, the presence of narrow, incised petals that are supplied by a single vascular trace and divided into at least three lobes is a conspicuous synapomorphy of tribe Schizomerieae (Barnes and Rozefelds, 2000). The monophyly of the tribe was resolved in one of several alternative topologies found by Hufford and Dickison (1992), and Bradford and Barnes (2001) confirmed it with an analysis based on a combination of morphological and molecular data. Incised petals also occur in *Gillbeea* F. Muell. (an unplaced genus within Cunoniaceae; Bradford *et al.*, 2004); however, in that genus the petals are bifid and have distinctive apical glands (Hoogland, 1960; Dickison, 1975). In *Ceratopetalum gummiferum* Sm. and some *Schizomeria* species there may be additional incisions dividing some or all the three major lobes (Matthews and Endress, 2002). The petals in the fossil are similarly narrow, supplied by a single vascular trace and divided into three primary lobes, each of which is further divided into three secondary lobes, like the most elaborate petals observed in *Ceratopetalum gummiferum*.

Several authors have studied the floral morphology and anatomy of Schizomerieae in detail (Hoogland, 1960; Dickison, 1975; Matthews *et al.*, 2001; Matthews and Endress, 2002). Extant members of the tribe are united by several morphological synapomorphies, including weakly heterogeneous wood rays, a nectar-producing floral disc that may be annular or segmented, a thecal connective extension on the anthers (Bradford and Barnes, 2001) and flattened dicolporate pollen with a discontinuous tectum.

The four extant genera of Schizomerieae are readily distinguished by their floral morphology (Barnes and Rozefelds, 2000). *Anodopetalum* has regularly 4-merous flowers with a shallow floral cup, petals with three acute teeth, long connective extensions (i.e. as long as the thecae, or longer), and a glabrous ovary with two partly fused styles (Barnes and Rozefelds, 2000). *Platylophus* has mostly 4-merous flowers (rarely pentamerous) with a shallow floral cup, petals with three lobes, the outer two of which are acute, whereas the middle may have a rounded apex or be acute, anthers with short connective extensions (i.e. shorter than the thecae), and a pubescent ovary with two free styles. *Schizomeria* species have 4- to 6-merous flowers with shallow floral cups, segmented (rather than annular) floral discs, petals with three (occasionally more) acute lobes, anthers with short connective extensions and glabrous ovaries with (usually) two free styles. *Ceratopetalum* species are characterized by 4- to 6-merous flowers with a semi-inferior ovary, and most lack petals. *Ceratopetalum gummiferum* is the only extant species with petals (Rozefelds and Barnes, 2002), but they are also present in some fossils assigned to *Ceratopetalum* (Holmes and Holmes, 1992; Barnes and Hill, 1999). In *Ceratopetalum gummiferum* the petals are divided into three to nine (usually five) lobes. Some *Ceratopetalum* species have anthers with connective extensions nearly as long as the thecae (like *Anodopetalum*), whereas others have shorter connective extensions (as in *Schizomeria* and *Platylophus*; Barnes and Rozefelds, 2000). Some species have glabrous ovaries, whereas others have pubescent ones (Barnes and Rozefelds, 2000).

Several features are shared by *Lacinipetalum* and the extant genera of Schizomerieae. These include incised petals, a floral disc and a connective extension in the anthers. *Lacinipetalum, Schizomeria*, *Platylophus* and *Anodopetalum* have shallow (palletiform) floral cups and superior ovaries. Anthers with short connective extensions are shared among *Lacinipetalum, Schizomeria*, *Platylophus* and some species of *Ceratopetalum*. Pubescent ovaries are present in *Platylophus*, some *Ceratopetalum* and *Lacinipetalum*, but only *Platylophus* and *Schizomeria* have free styles, as in *Lacinipetalum*. The highly divided petals of *Lacinipetalum* are most like those of *Ceratopetalum gummiferum*.

There are also several features of *Lacinipetalum* that are different from the extant Schizomerieae but still consistent with Cunoniaceae. For example, the number of sepals and petals typically varies from four to six (within single plants) in the Schizomerieae, but in *Ceratopetalum* the flowers are rarely up to 8-merous (Bradford *et al.*, 2004; BH 00081374). *Lacinipetalum* normally has eight to ten perianth parts. We suggest that although *Lacinipetalum* has twice the number of perianth parts typically observed in extant Schizomerieae, the same developmental control underlies the within-plant variation in merosity. Another feature that separates *Lacinipetalum* from the extant genera is the morphology of the pollen. In extant Schizomerieae, the pollen is dicolporate with a heterogeneous tectum (Hideux and Ferguson, 1976); however, in *Lacinipetalum* the pollen is tricolporate and the exine is punctate and homogeneous (Fig. 4B), as in *Davidsonia* (Fig. 4C), *Spiraeanthemum* A. Gray and some *Weinmannia* L. (Moar, 1993; Bradford and Barnes, 2001). The pollen found within the anthers and on the ovary hairs is similar to the dispersed tricolporate pollen grains that were attributed to Cunoniaceae from the Maastrichtian Lefipán Formation (Baldoni and Askin, 1993) and the early Palaeocene Salamanca and Bororó formations, Chubut (Archangelsky, 1973; Petriella and Archangelsky, 1975; Graham, 2010).

All previously described fossil flowers assigned to Schizomerieae have been placed within extant genera (Barnes *et al.*, 2001). The fossils *Ceratopetalum priscum* W.B.K. Holmes & F.M. Holmes and *Ceratopetalum wilkinsonii* (Ett.) W.B.K. Holmes & F.M. Holmes emend. Barnes & Hill are interpreted as flowers because they retain petals and the sepals are not expanded into obovate wings (Holmes and Holmes, 1992; Barnes and Hill, 1999). They are both distinguishable from *Lacinipetalum* by their pentamerous structure and the trifurcate petals, but it is intriguing that in *C. priscum* the sepals are lanceolate and have uncontracted bases like those of *Lacinipetalum*. Holmes and Holmes (1992) used this feature to distinguish *C. priscum* from other *Ceratopetalum.* Nonetheless, we concur that these fossils are attributable to *Ceratopetalum* and not *Schizomeria* or *Platylophus* based on the semi-inferior ovaries (Barnes and Hill, 1999). The only other fossil taxon with petals assigned to Schizomerieae is *Schizomeria tasmaniensis* R.J. Carpenter & A.M. Buchanan (Carpenter and Buchanan, 1993). This species is known from a single pentamerous flower with short-trifurcate petals that have acute lobes. The assignment to *Schizomeria* was justified by the pentamerous structure, size, and shape of the preserved floral organs. Among the significant features that Carpenter and Buchanan (1993) used as part of their justification for assigning the fossil to *Schizomeria* rather than *Platylophus* were the acute lobes of the trifurcate petals, as *Platylophus* has a rounded central lobe; however, we have not found that to be a reliable character (e.g. BH 46253; 53995). Nonetheless, the short trifurcate petals and pentamerous structure distinguish *S. tasmanensis* from *Lacinipetalum*.

*Tropidogyne pikei* K.L. Chambers, Poinar, & R.T. Bucklet from the Cenomanian of Burma was compared with Schizomerieae based on the pentamerous organization, inferior ovary, absence of petals, diplostemonous androecium, introrse anthers and ridges on the exterior of the hypanthium (Chambers *et al.*, 2010; Shi *et al.*, 2012). Although these states are found in Cunoniaceae, many are plesiomorphic for the tribe, whereas others are found across the rosid clade (Matthews and Endress, 2002). Furthermore, as noted by Chambers *et al.* (2010), *Tropidogyne pikei* lacks some synapomorphies of Schizomerieae, such as the broad torus (nectary disc) surrounding the gynoecium into which the stamens are inserted, the connective extension on the anthers and the hairs covering the ovary. Poinar and Chambers (2017) described a second species, *T. pentaptera* also from the Burmese amber that is even more like some apetalous *Ceratopetalum* species in the shape and venation of the calyx, the presence of a synovarious, bicarpellate, distylous gynoecium, and anthers with a connective extension. Originally, Chambers *et al.* (2010) suggested, based on the age of the fossils, that *Topidogyne* may be nested deep within the rosids and thus display a suite of plesiomorphic characteristics for the clade, a hypothesis that seems plausible given the work of Matthews *et al.* (2001). By contrast Poinar and Chambers (2017) proposed a relationship to *Ceratopetalum*, but they did not include a phylogenetic analysis, nor did they state the nature of the proposed relationship (e.g. nested within the apetalous clade, sister to the genus, or elsewhere in Cunoniaceae or Oxalidales). Because of the differences between the two species, we suggest that a critical evaluation of the phylogenetic position of these species requires their inclusion in a broad phylogenetic analysis of rosid floral characteristics, a task that is beyond the scope of this work.

### Phylogenetic analyses

The results of our total evidence analysis (Fig. 5) are congruent with the topology presented by Bradford and Barnes (2001). Independent analysis of the morphological data demonstrates that there is sufficient diversity in the Schizomerieae to resolve the relationships among the genera in the tribe based on morphology alone, and the topology obtained from parsimony analysis of morphological data agrees with that obtained from *rbcL* and *trnL-trnF* sequence data. We found strong support for the hypothesis that *Lacinipetalum* is sister to extant Schizomerieae, and that *Schizomeria* is sister to *Ceratopetalum*, *Anodopetalum* and *Platylophus*. This result was contrary to our initial hypothesis that the fossil might be more closely related to *Ceratopetalum* based on the similarity between the petals in *Ceratopetalum gummiferum* and *Lacinipetalum*. The drupaceous fruits of *Schizomeria* are developmentally different from the drupaceous fruits of *Davidsonia* (Doweld, 1998), suggesting that drupaceous fruits are not necessarily the ancestral state for the tribe. Additional data from fossil fruits of *Lacinipetalum* would be useful to further test the hypothesis that fleshy drupes are the primitive condition for the tribe.

### Paleoecology and paleobiogeography

Extant Schizomerieae have a restricted distribution (Fig. 1). *Ceratopetalum* (eight species) and *Schizomeria* (ten species) co-occur in the rainforests of eastern Australia (Campbell, 1923; Burges and Johnston, 1953; Baur, 1957; Hoogland, 1960; Binns, 1995; Rozefelds and Barnes, 2002; Crisp *et al.*, 2004; Boland *et al.*, 2006). Only one extant species of *Ceratopetalum, C. succirubrum* C.T. White, occurs outside of Australia today; in addition to Australia, it occurs in New Guinea and adjacent islands, including New Britain (Hopkins and Hoogland, 2002; Rozefelds and Barnes, 2002), where it grows in montane rainforests (Takeuchi, 1999a, b, 2003; Paul, 2011). By contrast, at least eight species of *Schizomeria* occur outside of Australia in lowland to montane rainforest and extend into secondary vegetation, scrub and the margins of savanna or alpine grassland in the Moluccas, New Guinea, the Bismarck Archipelago and the Solomon Islands (Hopkins, 2001; Hopkins and Hoogland, 2002; Paul, 2011). *Anodopetalum biglandulosum* A. Cunn. ex Hook.f. is endemic to the cool temperate forests of Tasmania (Barker and Brown, 1994), and *Platylophus trifoliatus* is endemic to the warm temperate riparian forests of the Cape floristic region of South Africa (Phillips, 1925; Dyer, 1951; Bond *et al.*, 1984). Despite the emerging fossil record of the Schizomerieae in Patagonia (Gandolfo and Hermsen, 2017; this paper), the tribe is conspicuously absent from South America today.

Barnes *et al.* (2001) reviewed the fossil record of Cunoniaceae and accepted only six occurrences of Schizomerieae (Table 3), all of which were from Australia. Together, those fossils confirm the presence of the tribe in Australia since at least the early Eocene (Fig. 1). Recently, Gandolfo and Hermsen (2017) reported the presence of *Ceratopetalum* fruits in the early Eocene (52 Ma) Tufolitas Laguna del Hunco in northwest Chubut, Argentina. This was the first report of fossil Schizomerieae outside of Australia and the oldest record of the tribe. That discovery and the flowers described here demonstrate that the group was more widespread in the past and probably extended across Antarctica during the warm early Eocene (Kooyman *et al.*, 2014; Gandolfo and Hermsen, 2017; this paper).

Crisp *et al.* (2004) suggested that the diversity of the Schizomerieae in Australia (*Ceratopetalum*, *Schizomeria* and *Anodopetalum*) has remained stable or possibly declined since the Oligocene as the total area of suitable rainforest habitat has decreased, a conclusion consistent with the fossil evidence (Kershaw and Sluiter, 1982; Barnes *et al.*, 2001; Carpenter *et al.*, 2004). With the Patagonian records, it now seems apparent that the diversity and geographical range of the tribe across the entire Southern Hemisphere has declined since the Palaeogene. The discovery of *Lacinipetalum* fossils in early Palaeocene deposits together with the recent discovery of *Ceratopetalum* in early Eocene deposits, both in southern South America (Gandolfo and Hermsen, 2017; this paper), and the distribution of *Platylophus* in South Africa (Don, 1830; Engler *et al.*, 1887; Phillips, 1925) suggest that the Schizomerieae originated somewhere in the widespread wet forests of Gondwana, perhaps in South America, and that the modern distribution of the tribe is a result of range contraction associated with post-Eocene cooling and drying in the Southern Hemisphere. Although Schizomerieae persist in South Africa, the pattern of extirpation from South America and survival in Australasia has been identified in several other rainforest lineages known from Palaeogene fossils in Patagonia, including ferns, conifers and angiosperms (e.g. Zamaloa *et al.*, 2006; Wilf *et al.*, 2009; Gandolfo *et al.*, 2011; Wilf, 2012; Hermsen *et al.*, 2012; Carvalho *et al.*, 2013; Kooyman *et al.*, 2014).

*Lacinipetalum* was part of a diverse community in a moist to humid temperate climate (Iglesias *et al.*, 2007). In the Salamanca Formation, conifers dominate the fossil wood assemblages, but angiosperms dominate in assemblages of leaf compressions, where they also constitute most of the species richness (Iglesias *et al.*, 2007; Escapa *et al.*, 2013). The *Lacinipetalum* flowers are the most abundant type of reproductive macrofossil at the PL-2 locality. Some of the groups identified based from the Palacio de los Loros localities include flowering plants in Akaniaceae, Fabaceae, Lauraceae, Menispermaceae, *Nothofagus* (Nothofagaceae), Sapindaceae, Urticaceae, the conifers *Agathis* and *Dacrycarpus* and the fern *Lygodium* (Schizaeaceae) (Iglesias *et al.*, 2007; Brea *et al.*, 2008; Escapa *et al.*, 2013; Quiroga *et al.*, 2016). The Las Flores locality, where two specimens of *Lacinipetalum* were found, is similarly angiosperm-dominated with rare conifers and ferns, but so far it has received little attention (Donovan *et al.*, 2016). Other lineages identified from various Salamanca Formation and other Palaeocene localities in Patagonia include Cheirolepidiaceae (Barreda *et al.*, 2012), Podocarpaceae, Cupressaceae (Ruiz *et al.*, 2017), cycads (Petriella, 1972), ferns and lycophytes (Archangelsky, 1973), Arecaceae (Romero, 1968; Petriella, 1972; Archangelsky, 1973; Futey *et al.*, 2012), Rhamnaceae (Jud *et al.*, 2017), Myrtaceae (Ragonese, 1980), Boraginaceae (Brea and Zucol, 2006), Elaeocarpaceae, Euphorbiaceae, Rutaceae and Cunoniaceae (*Weinmannioxylon* species) (Petriella, 1972). Together, these occurrences demonstrate that the flora included tropical and southern-wet forest elements; the biome probably resembled the temperate forests of Eastern Australia, where *Schizomeria* and *Ceratopetalum* are native today.

## CONCLUSIONS

The fossils described here are the second taxon of Danian flowers known from the Southern Hemisphere, after *Notiantha* Jud, Gandolfo, Iglesias & Wilf (Rhamnaceae), from a different locality in the Salamanca Formation (Jud *et al.*, 2017). *Lacinipetalum spectabilum* is the oldest reliable occurrence of crown-group Cunoniaceae, and it is most likely sister to the extant Schizomerieae because it shares some synapomorphies of that tribe. Recent Schizomerieae and some of their close relatives are typical of tropical and temperate forests in Australasia; thus, it is likely that *Lacinipetalum* grew in similar conditions and serves as an additional indicator of the development of moist to wet forest in Patagonia within no more than 2.5 million years of the end-Cretaceous extinction event. The discovery of *in situ* pollen supports the conclusion that dispersed tricolporate cunoniaceous pollen from the Maastrichtian and early Palaeocene deposits of Chubut, Argentina, do reflect survival of Cunoniaceae in South America across the Cretaceous–Palaeogene boundary. The occurrence of *Lacinipetalum*, together with the recent discovery that *Ceratopetalum* was present in Patagonia during the early Eocene, suggests that the Schizomerieae were widespread during the Palaeogene and may have originated outside Australia during the late Cretaceous or earliest Palaeogene.

## SUPPLEMENTARY DATA

Supplementary data are available at https://academic.oup.com/aob and consist of Appendix S1: Nexus file containing the DNA sequence alignment used in the phylogenetic analysis.

Supplementary Data

## FUNDING

This work was supported by the National Science Foundation (grant numbers DEB-1556136, DEB-0918932, DEB-1556666, DEB-0919071, DEB-0345750) and the Fulbright Foundation (M.A.G.).
